# Hybrid Chin Advancement: Combining Fat and Sliced Cartilage Grafts for Chin Augmentation During Rhinoplasty

**DOI:** 10.1007/s00266-024-04137-4

**Published:** 2024-06-10

**Authors:** Engin Selamioğlu, İsmail Küçüker

**Affiliations:** 1 Department of Plastic Reconstructive and Aesthetic Surgery, Haliç University, Istanbul, Turkey; 2 Plastic Reconstructive and Aesthetic Surgery, Prive Clinic, Istanbul, Turkey

**Keywords:** Cartilage graft, Chin augmentation, Fat grafting, Rhinoplasty

## Abstract

**Background:**

Facial balance significantly impacts aesthetics, particularly in the middle and lower thirds. Patients with chin retrusion often benefit from sagittal plane chin advancement in rhinoplasty, enhancing surgical outcomes and satisfaction.

**Objectives:**

This article presents a method for analyzing chin deformities and discusses a hybrid treatment approach to harmonize facial features, complementing rhinoplasty.

**Methods:**

The chin positions of patients treated by the senior author were assessed. A retrospective analysis included 49 patients with chin retrusion of 2.5–6 mm. Among them, 22 patients initially offered chin implants declined, leading to planned chin augmentation. Fat grafting was exclusively performed for 20 patients lacking sufficient cartilage. The "Hybrid Chin Advancement" technique involved supporting tissues beneath muscles with nasal septum cartilage and fat injections and tissues above muscles with fat injection alone.

**Results:**

Pre- and postoperative Legan angle measurements and chin advancements were compared across three groups. While preoperative Legan angles were statistically similar, postoperative Legan angles and advancement changes were significantly higher in the implant group (*p *< 0.0001). Comparing hybrid chin advancement and fat grafting groups, postoperative Legan angles and advancement changes were significantly higher in the hybrid chin group (*p* < 0.0001).

**Conclusions:**

Fat grafting suffices for mild advancements (~ 2 mm), while the hybrid chin method is effective for moderate advancements (~ 4 mm). For advancements exceeding 6 mm, implants or osseous genioplasty are optimal. Our study’s hybrid approach offers an easy, safe, and reliable method for achieving facial harmony in the lower two-thirds without compromising patient expectations.

**Level of Evidence IV:**

This journal requires that authors assign a level of evidence to each article. For a full description of these Evidence-Based Medicine ratings, please refer to the Table of Contents or the online Instructions to Authors www.springer.com/00266.

**Supplementary Information:**

The online version contains supplementary material available at 10.1007/s00266-024-04137-4.

## Introduction

Rhinoplasty is one of the most common aesthetic surgical procedures [1]. The middle part of the face is a vital structure, and the harmony of the midface and other anatomical structures should also be prioritized to make the aesthetic results satisfactory to the patient and the surgeon. Failure to examine the relationship between the face’s middle third and upper and lower thirds on the sagittal plane is a standard error in preoperative evaluations for rhinoplasty. Therefore, to improve the aesthetic outcome from the lateral profile beside the nose, surgeons must examine the chin, midface, and frontal region [2]. A previous study showed that the most common associated problem among patients who seek rhinoplasty is chin retrusion [3] and the recognition, evaluation, and treatment of chin abnormalities. These features often have a tremendous impact on facial appearance [4]. Evidence also shows that at least 25% of all rhinoplasty patients may require chin augmentation [5]. Different procedures can be used for such augmentation, including osseous genioplasty, fat grafting, osteocartilaginous grafts, alloplastic implants, and tissue fillers [6]. However, fillers and fat injections seem to be easier ways for chin augmentations; genioplasty and chin implants still provide better support concerning pogonion to augment the chin on the sagittal plane [7]. However, aside from its advantages, genioplasty is associated with more significant morbidity than other methods [8], and implants involve risks of some foreign bodies [9]. Considering their disadvantages, these methods are not the first choices for practitioners. When surgeons suggest chin augmentations in patients who are seeking rhinoplasty, the patients become anxious about increased edema of the chin, the existence of foreign bodies in their chins, and the additional costs of the surgery. Therefore, these patients will likely choose methods associated with less morbidity and lower costs.

Fat grafting alone is beneficial in mild chin retrusion but fails to give enough support for intermediate retrusion. As a result, for chin augmentation, we proposed combining the fat grafts with the cartilage grafts harvested during the rhinoplasty, and we have called this the “Hybrid Chin Advancement.” Hybrid refers to combining two different materials used for a single purpose. In the hybrid chin advancement concept, we combined two types of tissues to advance the chin on the sagittal plane. In the fiction, deep tissues (submuscular compartment) were supported by nasal septum cartilage plus fat injection, and superficial tissues (supramuscular compartment) were supported by fat injection only. Our study aims to evaluate the technique’s advantages and disadvantages, including a combination of fat grafting and modified cartilage grafting in patients who have poor chin projection and have undergone rhinoplasty. Clinical findings regarding the safety and complications were also evaluated.

**Fig. 1:**
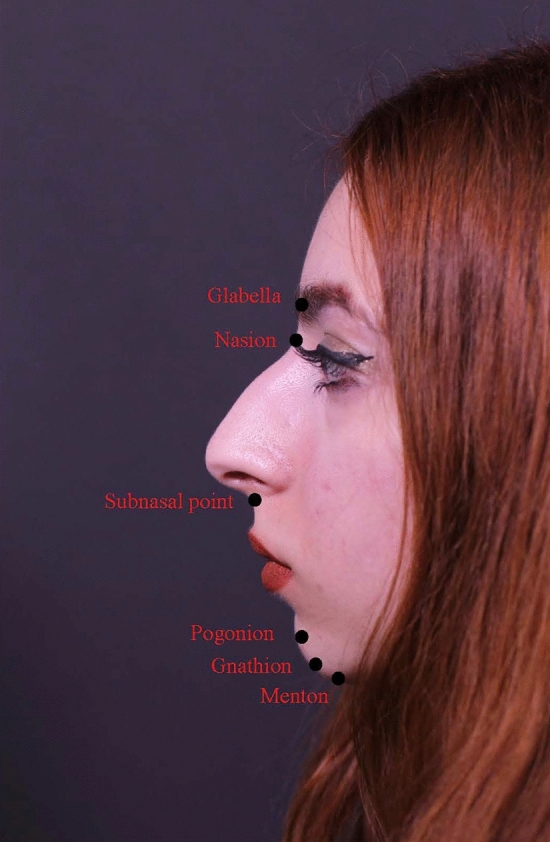
Schematization of pogonion, menton, and gnathion

**Fig. 2:**
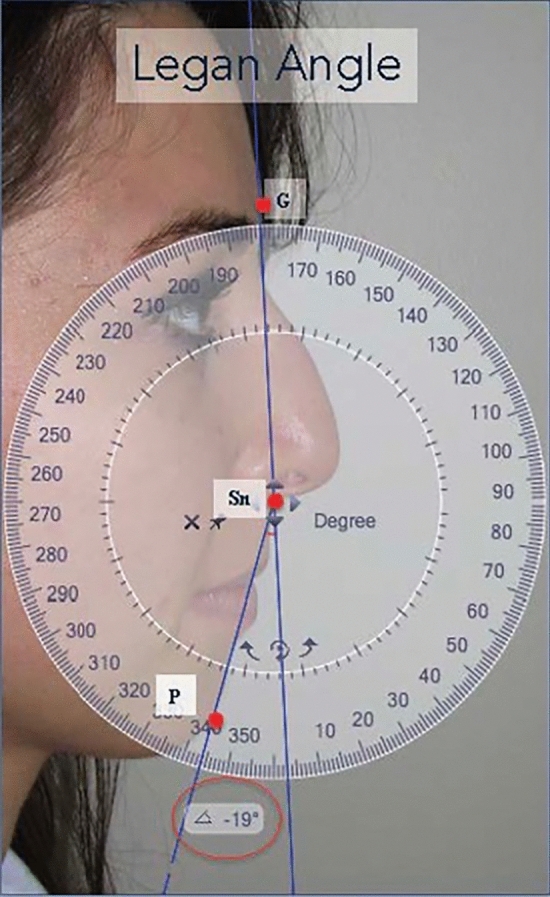
Measurement of Legan angle. G: glabella, Sn: subnasal, P: pogonion

The pogonion is the most anteriorly projecting point on the chin, while the menton is the most inferiorly projecting. The gnathion is the midpoint between the pogonion and the mention (Fig. 1). The dermis in this area is thick, measuring 2–2.5 mm in adults [5]. Below the dermis is a dense subcutaneous fat layer firmly attached to both the skin and the underlying musculature. The muscles of the chin, from the superficial to the deeper ones, include the depressor anguli oris, the depressor labii inferioris, and the mentalis muscles [5]. Under the muscle layer, there is also a deep fat layer with dense attachments to the underlying periosteum. There are many guidelines regarding the ideal chin projection on the sagittal plane. Legan proposed an ideal angle to evaluate facial convexity generally from 8° to 16°. Legan’s angle is measured along one line traced from the glabella to the subnasal point and another from the subnasal point to the pogonion [10] (Fig. 2).

## Methods

**Fig. 3:**
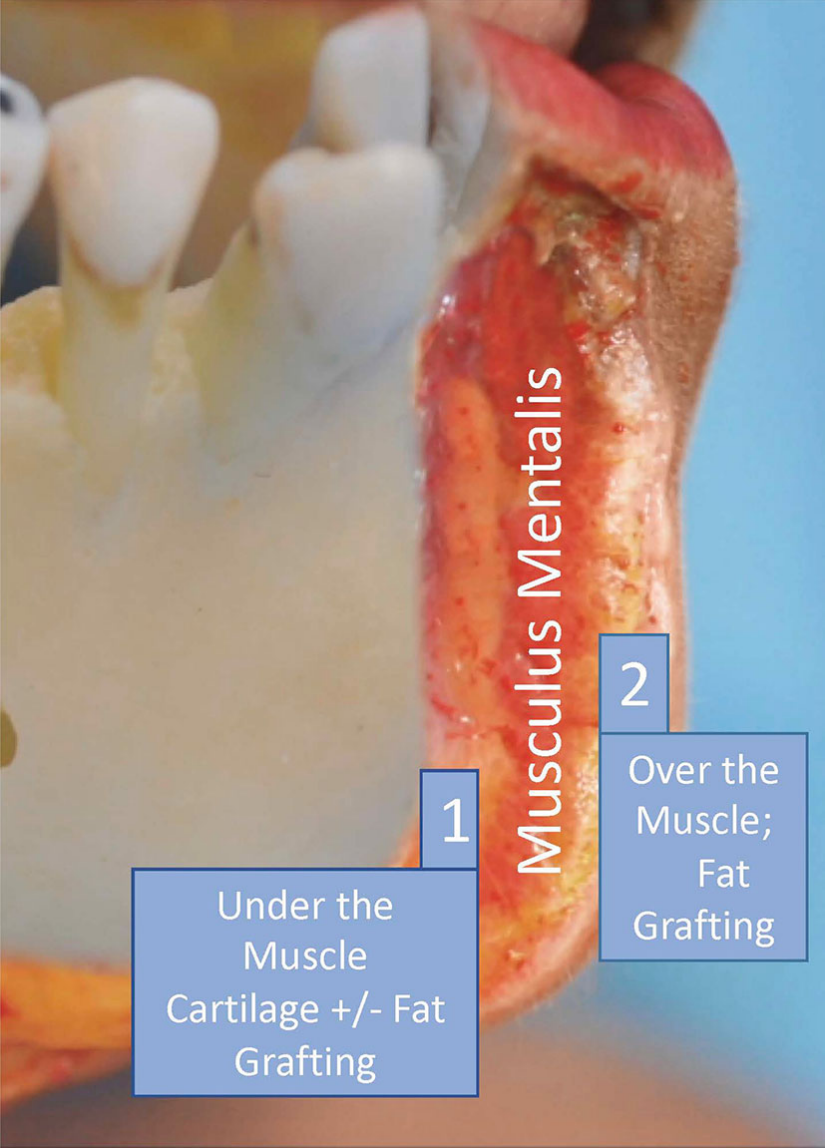
Conception of hybrid chin advancement

In this retrospective study, the senior author evaluated 533 patients who had undergone primary rhinoplasties from January 2018 to June 2020. The average follow-up period is 13 months. The patients included 201 males and 332 females. The age range of the patients is 20–37 years, and the average age is 27.75 years. Preoperative consent forms were obtained from all patients. Patients were consecutive. There were no criteria for inclusion/exclusion. Patient satisfaction was evaluated subjectively. Every patient was evaluated before and after surgery through a photographic analysis involving a three-dimensional photography analysis system (VECTRA®), and the Legan angle was measured on conventional photographs. This imaging system allows patients to preview the expected results of their procedure before committing to surgery. VECTRA 3D can be used before various plastic surgery procedures, including breast augmentation, body contouring, breast lift, chin augmentation, rhinoplasty, facelift, and neck lift. The authors applied chin augmentation in 151 of the 533 patients (28.3%). In 102 of these patients, the chin retrusion was less than 2.5 mm, and based on our previous clinical experience, fat grafting seemed to be enough for these mild retrusion cases. However, in 49 patients, the chin retrusion was more than 2.5 mm, and silicone implants were suggested for those cases. Of these 49 patients, seven accepted chin implants, while 42 did not want any artificial substances on their chins but were willing to have hybrid chin advancement. For this advancement concept, the tissues under the muscles were supported by nasal septum cartilage and fat injections. The tissues above the muscle were supported by fat injection only, and this procedure was called the “Hybrid Chin Advancement” (Fig. 3).

While performing each rhinoplasty procedure, required septal cartilage was resected subperichondrally for the cartilage grafts, and the lateral trochanteric area was the donor site of the fat grafts for all patients. The septal cartilage grafts were divided into smaller pieces and washed with 1–4 diluted povidone-iodine solutions. The tiny grafts were transferred to the chin from a 1 cm submental incision following a suitable pocket formation on the supraperiosteal plane. After the grafts had been placed, the deep fascia was sutured back by 6.0 polydioxanone sutures to avoid the migration of cartilage grafts. The fat grafts were centrifuged at 3000 rpm for 2 min, and the plasma-blood specimen was eliminated; then, fat grafts were injected into both superficial and deep planes by 21 gouge cannula from the lateral side of the chin (Video 1) [11]. The limit of the cartilage grafts was the harvested amounts, and the limit of the fat grafts was the soft tissue capacity. In 20 of the 42 planned hybrid chin advancement patients, the cartilage remaining at the end of the surgery was insufficient for the hybrid chin, and only fat grafting was applied to advance the chin. In 22 of the 42 patients, the cartilage amounts were sufficient to support the deep layers, and hybrid chin augmentation could be accomplished. Based on these findings, patients who need more than 2.5 mm advancement in the chin formed three groups: 7 had chin implants with 0.8 mm thickness in the sagittal plane, 20 had only fat injections, and 22 had hybrid chin augmentation. Patients required no special garments and were instructed to resume normal activities, including work, eating, etc., one day after surgery. For preoperative and postoperative day one, 1 g of cefazolin sodium was used as a prophylactic antibiotic. The Legan angles of all the patients were measured six months after surgery, and the results were also cross-checked by the three-dimensional photography analysis system (VECTRA®).

IBM SPSS Statistics 25 (IBM Corp. Released 2020. IBM SPSS Statistics for Windows, Version 25.0., Armonk, NY: IBM Corp.), MS Excel 2019, and Mann–Whitney U test were used for the statistical analysis and calculations. Before running the statistical tests for the angle, the variables were converted into "%" change between preoperative and postoperative values.

## Results

**Fig. 4:**
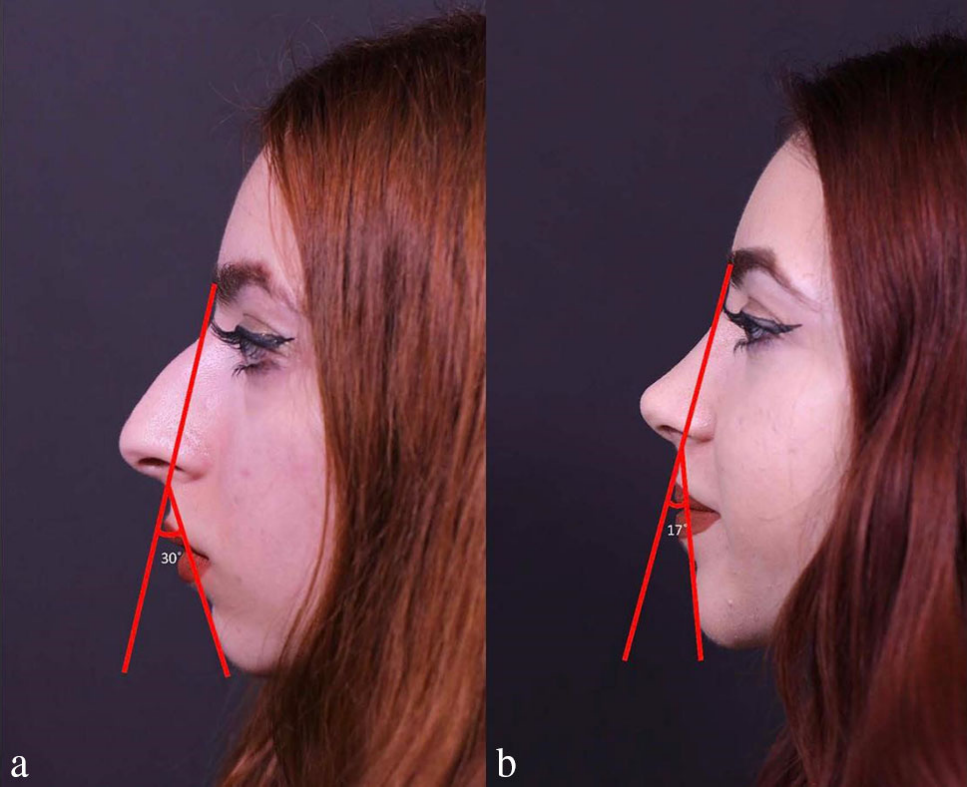
**a** Legan angle before hybrid chin advancement, **b** decreased Legan angle after hybrid chin advancement

**Fig. 5:**
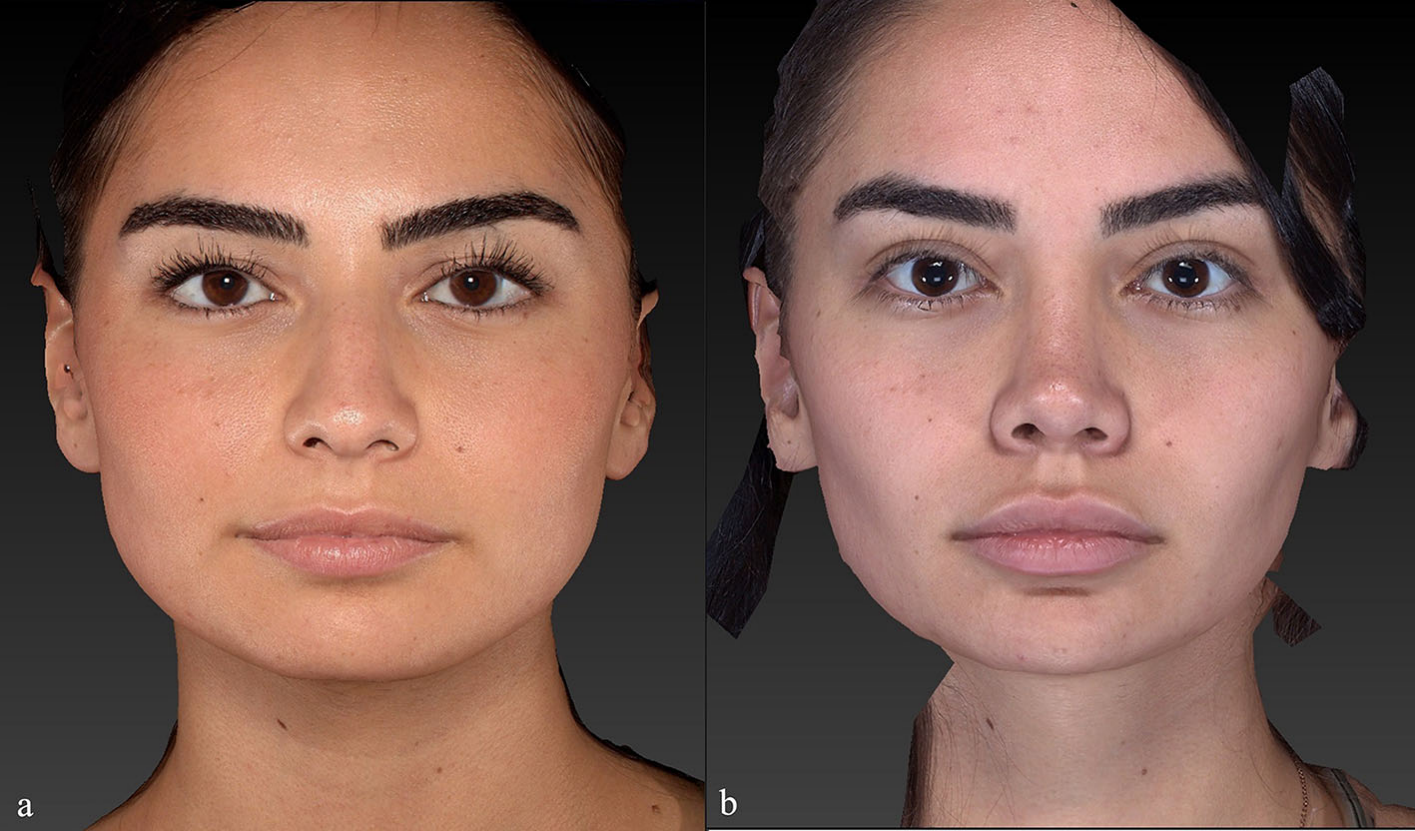
Anterior view of patient by VECTRA® **a** preoperative, **b** postoperative

**Fig. 6:**
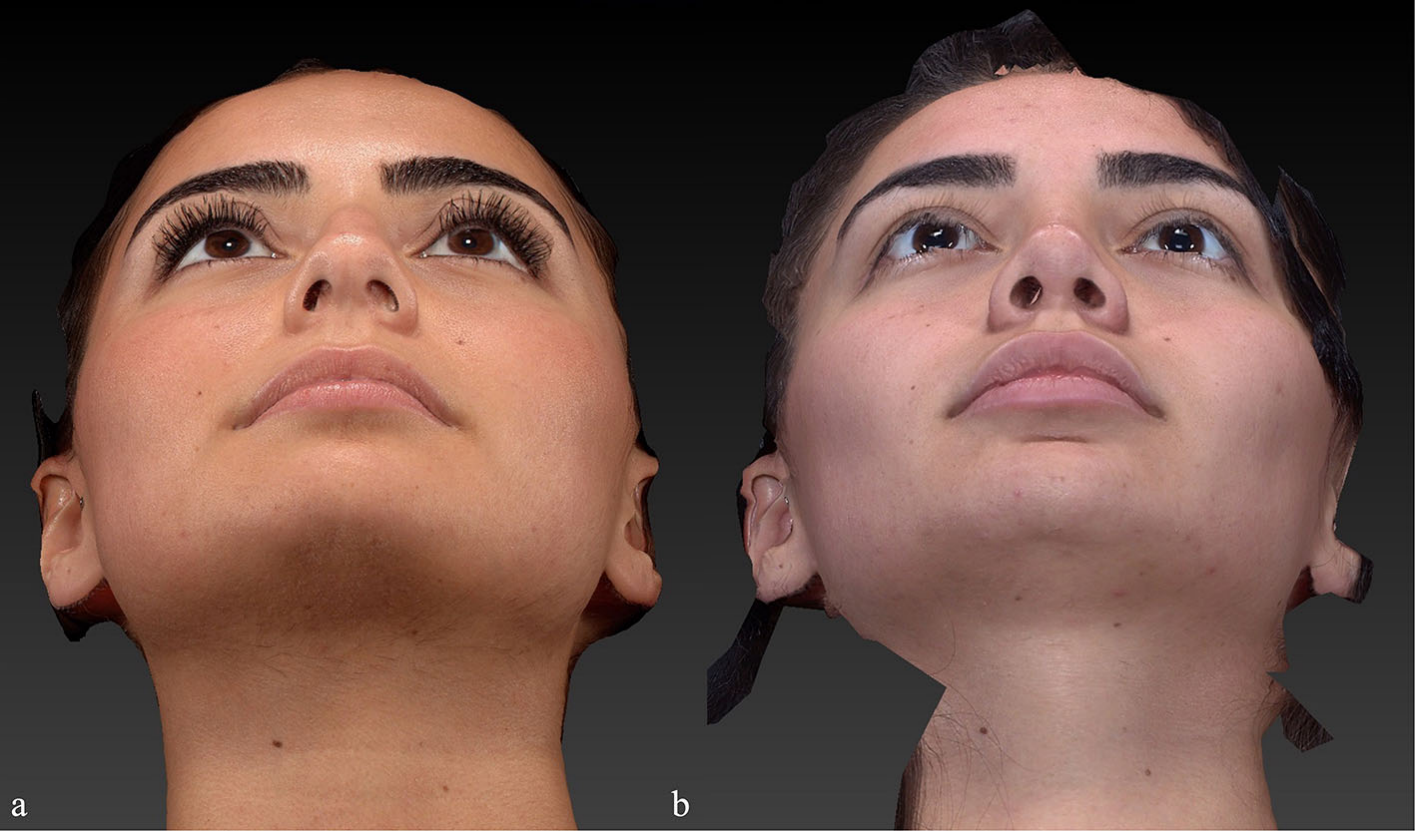
Basal view of patient by VECTRA® **a** preoperative, **b** postoperative

**Fig. 7:**
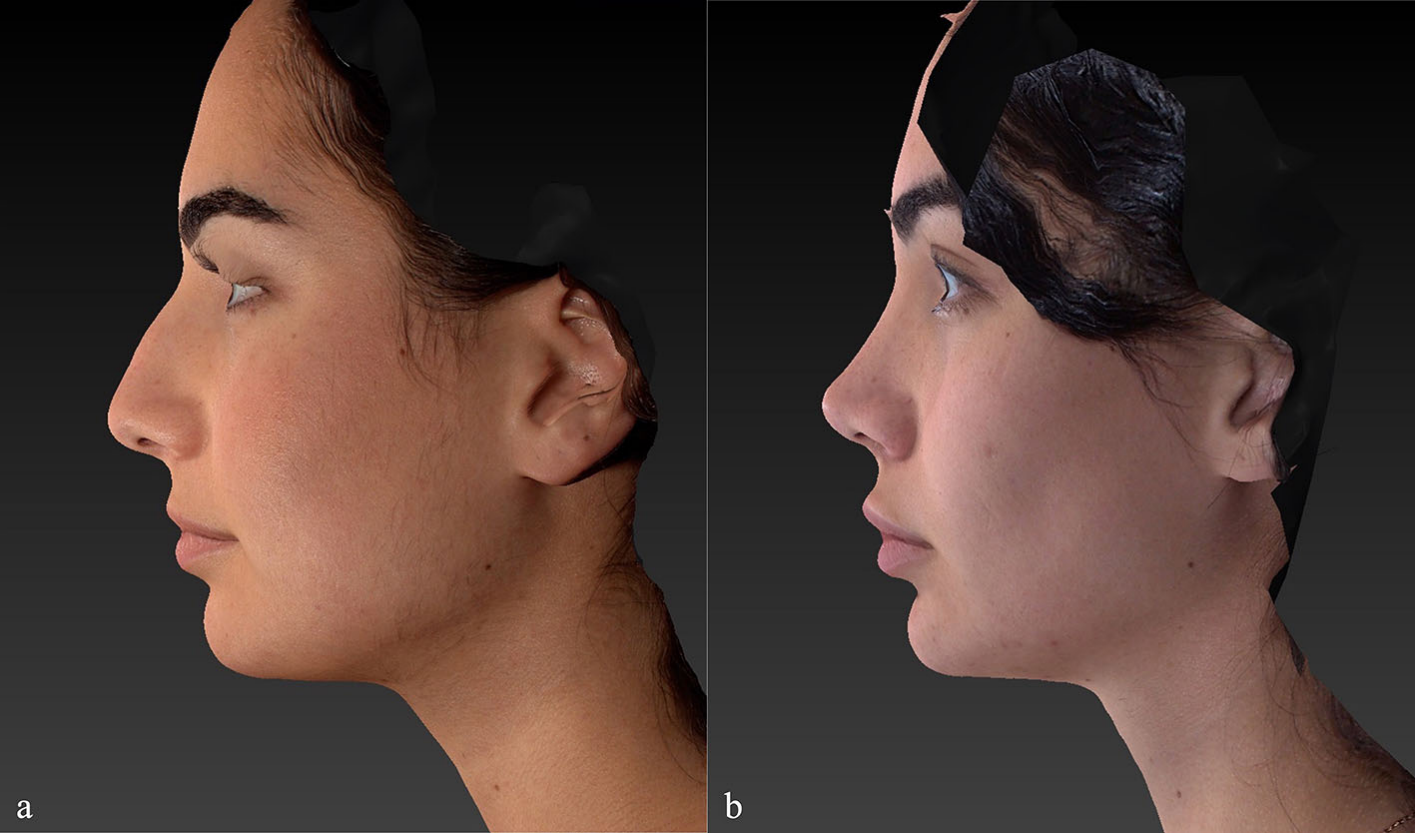
Left lateral view of patient by VECTRA® **a** preoperative, **b** postoperative

**Fig. 8:**
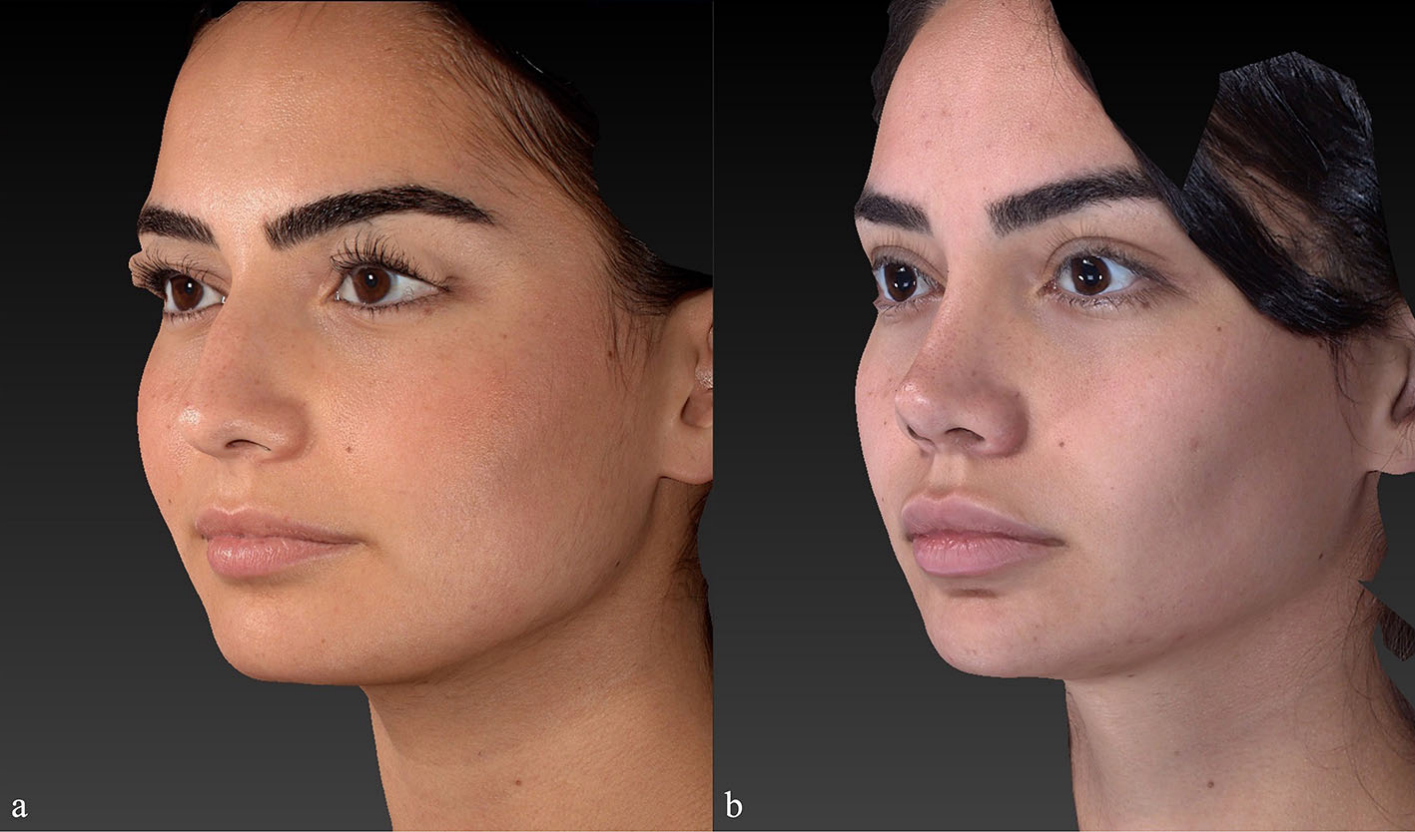
Left oblique view of patient by VECTRA® **a** preoperative, **b** postoperative

**Fig. 9:**
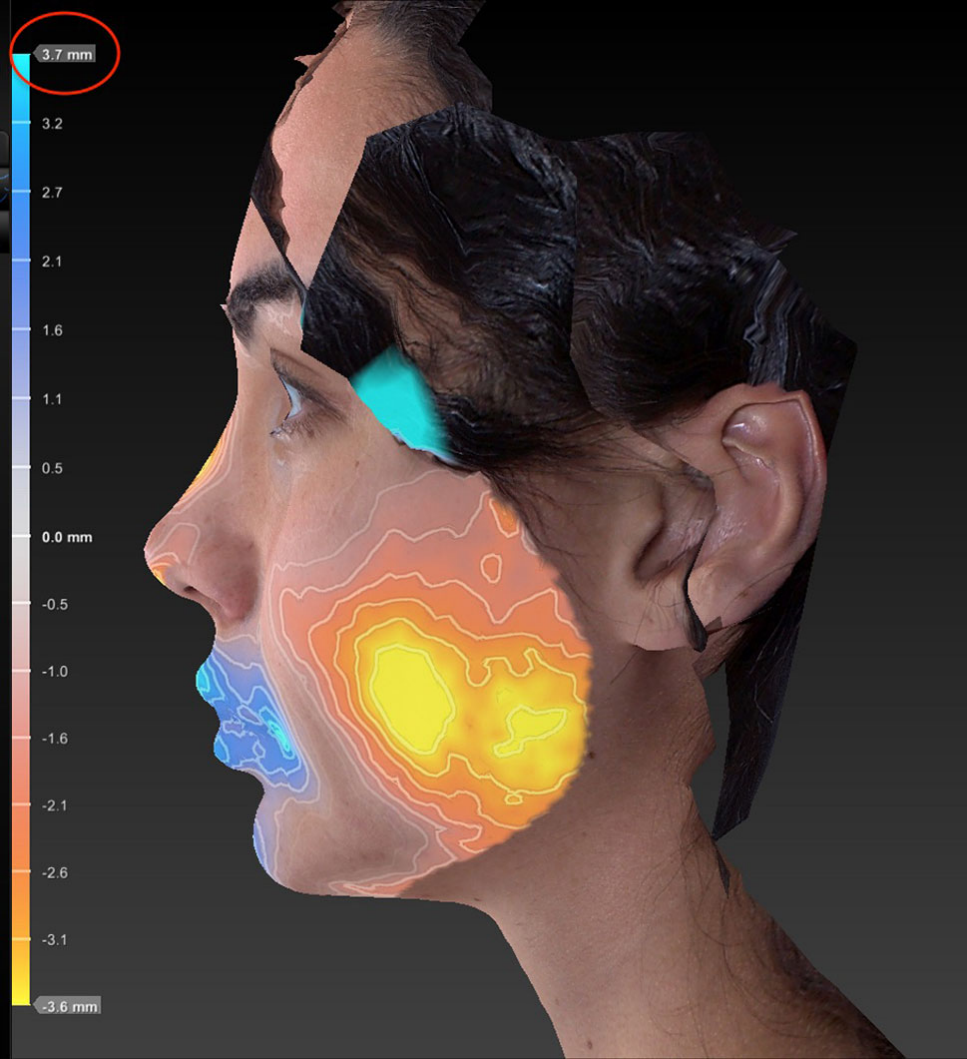
The red ring indicates increased chin projection after hybrid chin advancement

**Fig. 10:**
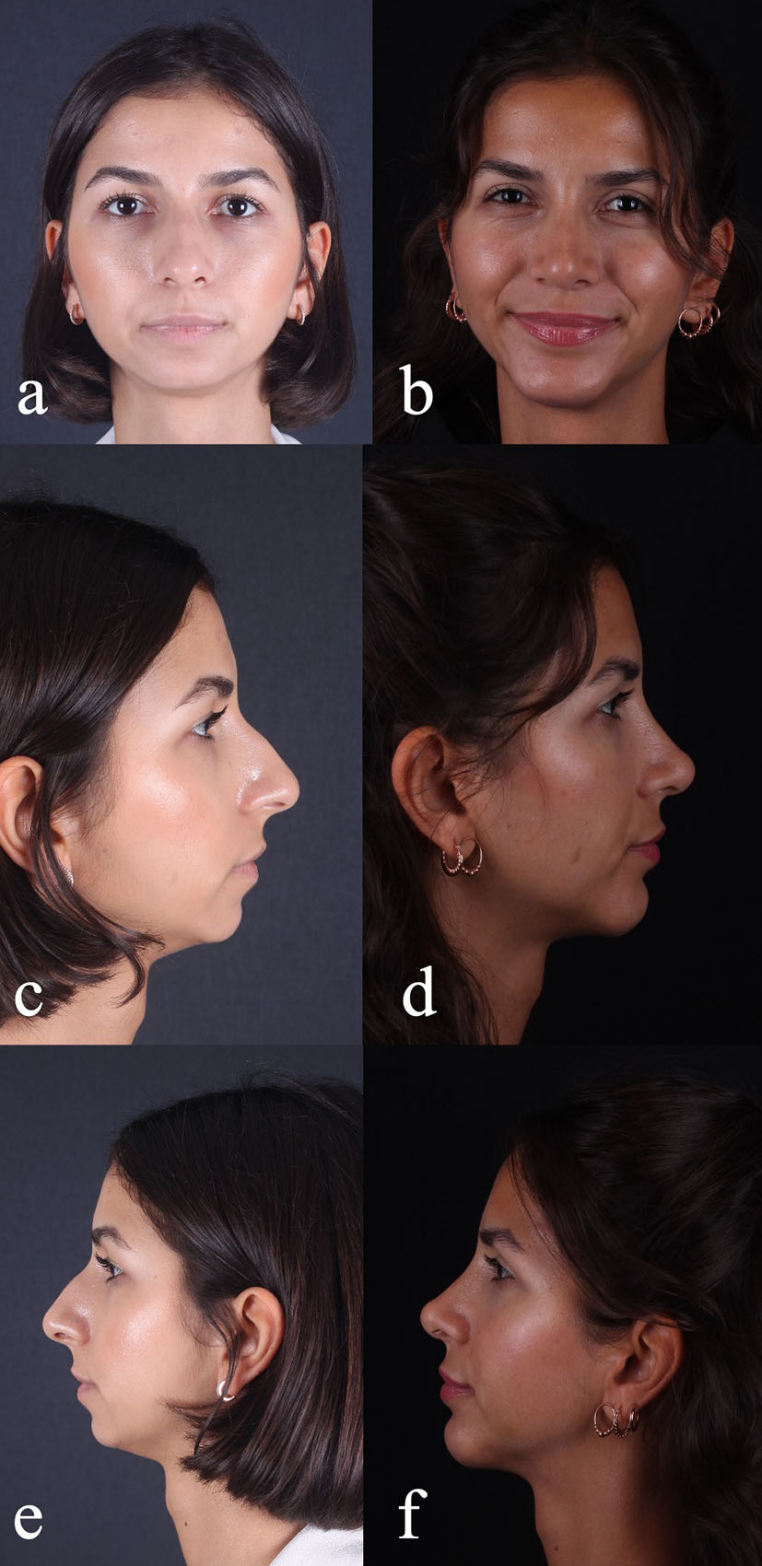
Views of patient with hybrid chin advancement, postoperative 1. Year follow-up **a** preoperative/anterior view, **b** postoperative/anterior view, **c** preoperative/lateral view/right side, **d** postoperative/lateral view/right side, **e** preoperative/lateral view/left side, **f** postoperative/lateral view/left side

**Fig. 11:**
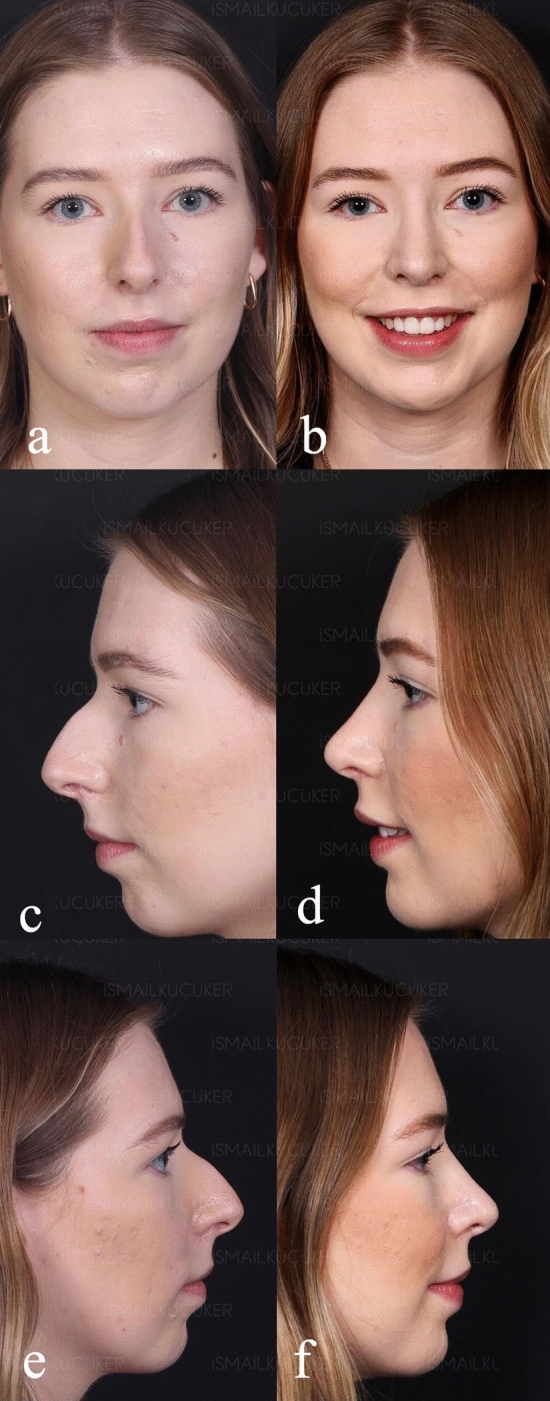
Views of patient with hybrid chin advancement, postoperative 1. Year follow-up **a** preoperative/anterior view, **b** postoperative/anterior view, **c** preoperative/lateral view/left side, **d** postoperative/lateral view/left side, **e** preoperative/lateral view/right side, **f** postoperative/lateral view/right side

Preoperative and postoperative Legan angle measurements and chin advancements, measured in mm, are summarized for all groups in supplemental Table 1. The preoperative Legan angles in the different groups were as follows: 23.20 (21–250) in the implant group, 23.80 (21–270) in the hybrid chin advancement group, and 23.30 (19–260) in the fat grafting group. When we compared the three groups, we could see that all groups’ preoperative Legan angles were statistically similar. When we examined the postoperative Legan and advancement changes among study groups, the changes were significantly higher (*p* < 0.0001) in the implant group [Legan changes: 8.50 (7–100) and advancement changes: 6.47 mm (6–6.6 mm)] when compared to both the hybrid chin group and the fat grafting group. The same data for the hybrid change group were as follows: Legan changes: 6.10 (5–70) (Fig. 4a, b) and advancement changes: 3.8 mm (3.1–4.4 mm) (Figs. 5a, b, 6a, b, 7a, b, 8a, b, 9) and for the fat grafting group they were the following: Legan changes: 4.00 (2–50) and advancement changes: 2.4 mm (1.9–3.1 mm). Also, when we compared the changes in the hybrid chin advancement and fat grafting groups, the postoperative Legan angles and advancement changes were significantly higher in the hybrid chin group (*p* < 0.0001) (Table 1). The mean follow-up period of the patients was 31 months (7–36 months), and the implant group encountered no complications during follow-ups. However, in three patients in the hybrid chin advancement group (13.6%), the cartilage grafts became palpable at the mental incision, and palpable cartilage was excised under local anesthesia. Two patients in the hybrid chin advancement (9%) and three in the fat grafting group (15%) complained about mild asymmetry, and six months after surgery, secondary fat grafting was applied to their chins, again under local anesthesia. Two patients’ postoperative 12-month follow-up views with hybrid chin advancement figures have been visibly seen (Figs. 10, 11).

**Table 1:** Shows the effect of different chin advancement techniques that are done during primary rhinoplasty

Surgical procedure	Number of patients	Preoperative Legan angle	Postoperative Legan angle	Change in Legan angle	Advancement in mm
Silicon ımplant (8 mm)	7	23, 2^+^	14, 7	8, 5*	6, 47**
Hybrid chin	22	23, 8^+^	17, 7	6, 1*	3, 8**
Fat grafting	20	23, 3^+^	16, 2	4, 0	2, 4**

^+^ indicates the preoperative legan angles were similar in between all groups (*p* > 0,1)

*, ** indicated the changes all among three groups are statistically significant (*p* < 0,0001)

## Discussion

A well-positioned chin is an aesthetically pleasing feature in men and women [12]. When evaluating the lower part of the face, a careful examination is of the most significant importance for maintaining a youthful, natural, and attractive appearance. However, the mentum is a complex area to evaluate and treat due to the number of structural variables, which include the length of the mandible, the position of the mandibular angle, the height and width of the chin, the thickness of the skin, the subcutaneous tissue, and the cervicomental and labiomental angles. As a result, it is crucial to evaluate the asymmetries of the face and the balance between the midface and lower face before any facial procedure is undertaken [13]. Therefore, plastic surgeons should add chin surgery procedures to rhinoplasty procedures. Different surgical and non-surgical procedures are used for augmentation of the chin; these include osseous genioplasty, fat grafts, osteocartilaginous grafts, alloplastic implants, and tissue fillers [6]. Osseous genioplasty and alloplastic implants often frighten patients, and there is no doubt that these are costly procedures with increased risks of morbidity. Mild-to-moderate microgenic patients may not need osteotomies or implants, and augmentation mentoplasty can be sufficient for balancing the facial profile. In the literature, the use of the nasal hump or septal cartilage has been described by Aufricht [14]. The iliac crest, cranium, and tibia are also popular donor sites for bone grafts for chin augmentation. According to the literature, harvesting from the oral cavity, such as the retromolar and ramus bones, is also frequently performed [15]. However, these procedures are complex and have high morbidity rates for patients. According to many authors, autologous cartilage and fat grafts represent excellent materials as a first choice for tissue augmentation [16, 17]. However, the major problem in using biological materials is resorption potential, and the main advantages of cartilage grafts are that they are viable even with poor blood supply and minimal resorption rate [18]. The long-term results of fat grafting are often disappointing because of unpredictable partial absorption of the fat grafts. Several studies have reported resorption rates of 30–70% within a year [19]. Therefore, according to the literature, serial fat injections for chin augmentation may be needed [20]. The patients in our study were evaluated in the sixth month to measure the Legan angles after the surgery, and we did secondary fat grafting in five patients to fix mild asymmetries. Since the biologic grafts have a risk of infection in the early postoperative period because of the alloplastic implants, we washed the cartilage tissue with 1–4 diluted povidone-iodine solution. We applied pre and single-dose postoperative antibiotics 12 h after the surgeries. We did not face any infections after surgery. Also, after completing the early period, in long-term follow-ups, we did not see late-onset infections due to the integration and increased vascularity of the biologic grafts [21].

One of the significant problems with osseous genioplasty is nerve injury [22]. To minimize the risk of paresthesia, surgeons must remember that the inferior alveolar nerve begins as inferior to the mental foramen, while the loop is anterior to it [23]. In our study, the core of augmentation was over the deep plane fat compartment, so the primary advantage of our procedure is that there is no risk of nerve injury or mental muscle dysfunction. Autologous fat grafting of the facial fat compartments has been shown to improve facial aesthetics. However, despite the increased use of fat grafting to fill the aging face, few reports have described fat grafting as a means for chin augmentation [9]. Fat augmentation of the chin can restore volume loss related to aging and soften the marionette lines that are difficult to correct with traditional surgical techniques such as osseous genioplasty or implants. In such cases, autologous fat grafting facilitates the asymmetries that cannot be corrected for the lower face using the abovementioned techniques.

Fat grafts are effective in mentum augmentation, but anterior projection gains with fat grafts are often around 2.4 mm. In hybrid chin advancement techniques, this gain can increase to 4.4 mm, depending on the amount of cartilage grafts. Evaluation of the Gonzalez-Ulloa line, the Silver line, and the Legan angle should serve only as reference planes because the analysis of facial aesthetics is complex [24]. The lower lip should usually have a prominence similar to the chin projection. Excessive lower lip projection or mentum can deepen the labiomental crease [25]. Therefore, in 11 patients, we also injected into a labiomental crease to achieve a better aesthetic outcome. In the literature, some reports show that respiratory mucoceles or atypical cystic formations may develop after using a nasal osteocartilaginous graft [26].

The cause of this clinical manifestation can be explained by the presence of epithelial cells that have not been appropriately removed from the resected osteocartilaginous graft. However, in our study, no early or late complications were observed in any of the patients included. The patients who planned hybrid chin advancement but could not harvest adequate cartilage grafts were only injected fat grafts, and these patients should have been informed about the ear cartilage graft beforehand. Also, fat and cartilage grafting may be required in the long term compared to alloplastic implants. The differences of some chin augmentation techniques are shown in Table 2. Although the amount of cartilage to be removed from patients is unknown, cartilage implantation is decided intraoperatively. This is one of the study’s limitations, and the study’s sample size, retrospective design, and follow-up time are other limitations. However, this method shows that it is possible to use autologous implants as an alternative to alloplastic implants. No extra donor area is required when performed simultaneously with the rhinoplasty operation. Septal cartilage grafts can be shaped more easily than costal cartilage grafts. Cartilage septum implantation with fat injection ensures long-term permanence.

**Table 2:** Shows the difference of different chin advancement techniques that are done during primary rhinoplasty

Silicone ımplant	Fat grafting	Hybrid chin
Allogen	Autogen	Autogen
~ 4 cm incision	No incision	~ 5 mm incision
No resorption	Resorption	Resorption
Immediate results	Late results	Immediate results

## Conclusions

The proper position of the chin and the nose plays a vital role in the aesthetic appearance of the lower two-thirds of the face. The fat grafting will be adequate in patients who need mild advancement of around 2 mm, and hybrid chin will serve well when moderate advancement, such as 4 mm, is needed. However, if a patient needs more than 6 mm, the best technique to meet the expectations is implants or osseous genioplasty. The hybrid method mentioned in our study provides an easy, safe, and reliable way to obtain the harmony of the lower two-thirds of the face.

### Supplementary Information

Below is the link to the electronic supplementary material.

Supplementary file1 (DOCX 13 KB)

Supplementary file2 (MP4 26878 KB)
