# Supplementary material for: Hybrid Chin Advancement: Combining Fat and Sliced Cartilage Grafts for Chin Augmentation During Rhinoplasty
Source: Aesthetic Plast Surg. 2024 Jun 10;48(14):2625–33. doi: 10.1007/s00266-024-04137-4 (PMC11281964; doi:10.1007/s00266-024-04137-4)
Supplement: Supplementary file 1 — Supplementary file1 (DOCX 13 KB) [file 266_2024_4137_MOESM1_ESM.docx]

| Surgical Procedure | Number of Patients | Preoperative Legan Angle | Postoperative Legan Angle | Difference in Legan Angle | Advancement in mm |
| --- | --- | --- | --- | --- | --- |
| Silicon Implant  (8 mm) | 7 | 23,2 ^+^ | 14,7 | 8,5 ^*^ | 6,47 ^**^ |
| Hybrid Chin | 22 | 23,8 ^+^ | 17,7 | 6,1 ^*^ | 3,8 ^**^ |
| Fat Grafting | 20 | 23,3 ^+^ | 16,2 | 4,0 ^*^ | 2,4 ^**^ |

Supplemental Table 1; Shows the effect of different chin advancement techniques that are done during primary rhinoplasty. “+” Preoperative Legan Angles were similar in between all groups (p>0,1) *,**; changes all among three groups are statistically significant (p<0,0001)
